# Unraveling the Potential Underlying Mechanisms of Mild Behavioral Impairment: Focusing on Amyloid and Tau Pathology

**DOI:** 10.3390/cells13131164

**Published:** 2024-07-08

**Authors:** Efthalia Angelopoulou, Anastasia Bougea, Alexandros Hatzimanolis, Nikolaos Scarmeas, Sokratis G. Papageorgiou

**Affiliations:** 1 1st Department of Neurology, Aiginition University Hospital, National and Kapodistrian University of Athens, Vasilissis Sofias Street 72-74, 11528 Athens, Greece; angelthal@med.uoa.gr (E.A.); ns257@cumc.columbia.edu (N.S.); sokpapa@med.uoa.gr (S.G.P.); 2 1st Department of Psychiatry, Aiginition University Hospital, National and Kapodistrian University of Athens, Vasilissis Sofias Street 72-74, 11528 Athens, Greece; alhatzi@med.uoa.gr

**Keywords:** mild behavioral impairment, neuropsychiatric symptoms, mild cognitive impairment, dementia, Alzheimer’s disease, amyloid pathology, tau pathology, preclinical AD, prodromal AD

## Abstract

The emergence of sustained neuropsychiatric symptoms (NPS) among non-demented individuals in later life, defined as mild behavioral impairment (MBI), is linked to a higher risk of cognitive decline. However, the underlying pathophysiological mechanisms remain largely unexplored. A growing body of evidence has shown that MBI is associated with alterations in structural and functional neuroimaging studies, higher genetic predisposition to clinical diagnosis of Alzheimer’s disease (AD), as well as amyloid and tau pathology assessed in the blood, cerebrospinal fluid, positron-emission tomography (PET) imaging and neuropathological examination. These findings shed more light on the MBI-related potential neurobiological mechanisms, paving the way for the development of targeted pharmacological approaches. In this review, we aim to discuss the available clinical evidence on the role of amyloid and tau pathology in MBI and the potential underlying pathophysiological mechanisms. Dysregulation of the hypothalamic–pituitary–adrenal (HPA) axis, disruption of neurotrophic factors, such as the brain-derived neurotrophic factor (BDNF), abnormal neuroinflammatory responses including the kynurenine pathway, dysregulation of transforming growth factor beta (TGF-β1), epigenetic alterations including micro-RNA (miR)-451a and miR-455-3p, synaptic dysfunction, imbalance in neurotransmitters including acetylcholine, dopamine, serotonin, gamma-aminobutyric acid (GABA) and norepinephrine, as well as altered locus coeruleus (LC) integrity are some of the potential mechanisms connecting MBI with amyloid and tau pathology. The elucidation of the underlying neurobiology of MBI would facilitate the design and efficacy of relative clinical trials, especially towards amyloid- or tau-related pathways. In addition, we provide insights for future research into our deeper understanding of its underlying pathophysiology of MBI, and discuss relative therapeutic implications.

## 1. Introduction

In the 21st century, dementia is one of the most significant challenges that healthcare systems face worldwide. Given the demographic shift resulting from an aging population and the concomitant increasing life expectancy, dementia has emerged as the “new epidemic” and a global public health priority [1]. It currently affects more than 50 million people globally, while its prevalence is estimated to approximately triple by 2050 [2]. Dementia is a chronic, progressive syndrome encompassing a heterogeneous group of diseases, characterized by cognitive decline and functional impairment in daily activities, resulting in a profound impact on patients, families, caregivers, and society [3]. The most common cause is Alzheimer’s disease (AD), accounting for approximately 60–70% of all cases [4], followed by vascular dementia (VD), mixed forms, dementia with Lewy bodies, Parkinson’s disease (PD) dementia (PDD), and frontotemporal dementia (FTD) [2]. AD is characterized by a continuum, marked by an extended preclinical phase. Subjective cognitive decline (SCD) describes the self-reported cognitive complaints accompanied by cognitive performance within normal limits, while mild cognitive impairment (MCI) represents an early phase of cognitive decline, characterized by objective abnormalities in cognitive testing, while the patient maintains functional independence [5].

AD is considered a multifactorial neurodegenerative disease, with both genetic and environmental factors contributing to its development. The exact pathophysiological mechanisms underlying AD pathogenesis and progression remain obscure. However, several cellular and molecular processes are implicated in its development, including abnormal protein aggregation, mitochondrial impairment, neuroinflammation, oxidative stress, excitotoxicity, synaptic dysfunction, epigenetic modification, lysosomal dysfunction, dysregulated apoptotic mechanisms and proteasomal impairment [4,6]. The classical neuropathological hallmarks of AD constitute the abnormal deposition of extracellular senile plaques consisting of amyloid-beta (Aβ), and intracellular neurofibrillary tangles (NFTs) consisting of phosphorylated tau protein [6]. These pathological alterations have long been the primary focus of research efforts aiming to understand the underlying molecular mechanisms of the neurodegenerative process in AD, as well as to develop appropriate biomarkers and therapeutic interventions [4]. In this regard, the detection of amyloid or tau pathology via fluid or neuroimaging biomarkers in non-demented individuals [in the cerebrospinal fluid (CSF), blood or positron-emission tomography (PET)] surpassing the pathological levels has been consistently associated with a higher risk of cognitive decline, MCI or dementia [7,8]. However, CSF or PET examinations are relatively time-consuming, invasive, costly, and often inaccessible, necessitating the need for the development of inexpensive and convenient markers for more accurate and effective risk stratification [9].

There is no available disease-modifying therapy for AD able to reverse or halt the neurodegenerative process in the brain. The failures of clinical trials of potential therapies in AD have been attributed to the inadequate identification of the early stages of the disease, insufficient recruitment, and our gaps in understanding the complex biological mechanisms [10]. However, early diagnosis and detection of individuals at risk is of paramount importance, allowing for timely interventions that may slow disease progression at least in a proportion of patients [11]. Notably, as amyloid-targeting monoclonal antibodies such as lecanemab become clinically available, early AD detection and the development of appropriate clinical markers of AD-related neurodegeneration becomes even more crucial [9].

Even though cognitive impairment is the core clinical feature of dementia, neuropsychiatric symptoms (NPS), including mood disturbances, apathy, and psychotic features are very common, affecting up to 97% of patients with AD [12]. NPS in AD dementia have been associated with worse quality of life, functional impairment, poorer cognitive outcomes, increased risk of institutionalization, higher mortality, more severe caregiver burden as well as more rapid progression of the disease [13,14,15]. Notably, NPS may also appear during the pre-symptomatic stages of cognitive decline. Over half of patients encounter NPS prior to receiving a diagnosis of dementia, including approximately one-third of those developing AD, with the most frequent ones being depressive symptoms and irritability [16]. Accumulating evidence suggests that the emergence of NPS among non-demented individuals in later life, including depressive and anxiety symptoms, compulsive behaviors, irritability, psychotic manifestations, loss of empathy and apathy, might increase the risk of cognitive decline, and the progression of MCI to dementia [17,18]. In this context, NPS in preclinical AD have recently attracted increasing attention, and led to the concept of mild behavioral impairment (MBI) [19]. MBI refers to the emergence of sustained NPS in older individuals with normal cognition, SCD or MCI, which represent an at-risk state for dementia. These NPS may involve alterations in behavior, personality, mood, thought and motivation, and can precede the onset of cognitive decline. In this context, MBI can be considered as a complementary condition to MCI, and it may serve as a convenient prodromal clinical marker and risk syndrome of dementia, contributing to a more accurate selection of at-risk individuals [19].

However, the concept of MBI is relatively new, and its characterization requires further investigation. The boundaries between mild neuropsychiatric changes attributed to normal changes or a current psychiatric condition might be sometimes vague. Elucidating the underlying neuropathology of MBI would be of paramount importance towards our better understanding of the clinical entity of MBI itself, but also the neurobiological mechanisms related to its conversion to cognitive decline. Unravelling the cellular and molecular mechanisms of the relationship between behavioral and cognitive impairment during the preclinical and prodromal phases of dementia will aid in delineating the role of amyloid deposition, tau accumulation and other related mechanisms in the trajectory of MBI, and pave the way for future advancements in personalized and targeted preventive and therapeutic strategies. Some of the practical and clinical consequences of this clarification would be that the efficacy of preventive measures for dementia targeting NPS might depend on the burden of amyloid or tau pathology, and patients can be better informed about prognosis. Furthermore, the effectiveness of therapeutic interventions targeting amyloid or tau might also differ, depending on the presence of NPS symptoms. This knowledge would facilitate the design of clinical trials, risk stratification and pool of at-risk individuals, thereby yielding significant impact on a both individual and public health level in the field of dementia prevention and care.

In this regard, a growing number of evidence suggests that MBI is associated with alterations in structural and functional neuroimaging studies, higher neurofilament light chain levels that are indicative of axonal damage, higher genetic predisposition to clinical diagnosis of AD, as well as AD-related biomarkers assessed in the blood, CSF, and PET. Neuropathologically, MBI has also been related to a neuropathologically confirmed diagnosis of AD [20], implying that both amyloid- and tau-related mechanisms may be critically involved in its development and link with AD dementia.

Although the general aspects and genetic background of MBI, as well as the association between AD biomarkers and NPS in preclinical AD have been already reviewed elsewhere [21,22,23,24], there is no recent review specifically focusing on the potential underlying mechanisms of MBI. In this review, we aim to explore the (i) clinical evidence on the role of amyloid and tau pathology in MBI based on studies using AD biomarkers, (ii) the potential neurobiological mechanisms of MBI, with an emphasis on amyloid and tau pathology, and(iii) potential therapeutic implications. For this purpose, we first introduce the readers to the concept of MBI, regarding its definition, clinical features, assessment methods and epidemiology, as well as the role of amyloid and tau pathology in the NPS in AD dementia. The main body of our review is structured into the following sections based on the aims of our work: (i) clinical evidence on the potential role of amyloid pathology in MBI, (ii) clinical evidence on the potential role of tau pathology in MBI, (iii) proposed amyloid- and tau-related underlying pathophysiological mechanisms of MBI, and (iv) potential therapeutic implications. Then, we discuss the available literature evidence to additionally provide insights for future research into our deeper understanding of the underlying pathophysiology of MBI, and discuss relative therapeutic implications.

## 2. Materials and Methods

We followed a systematic approach for the literature search. To explore the clinical evidence on the role of amyloid and tau pathology in MBI based on studies using AD biomarkers, we searched the MEDLINE and Scopus databases for cross-sectional and longitudinal clinical studies written in English, investigating the potential role of amyloid or tau pathology in MBI, published until March 2024. We used the following keywords in various combinations: “mild behavioral impairment”, “MBI”, “neuropsychiatric symptoms”, “NPS”, “psychiatric”, “behavioral”, “mild cognitive impairment”, “MCI”, “subjective cognitive decline”, “SCD”, “normal cognition”, “cognitive impairment”, “cognitive decline”, “preclinical”, “prodromal”, “dementia”, “Alzheimer’s disease”, “amyloid”, “amyloid-beta”, “Aβ”, “Aβ42”, “tau”, “p-tau”, “t-tau”, “Alzheimer’s disease biomarkers”, “AD biomarkers”, “neurodegeneration” and “neurodegenerative”. Based on the definition of MBI, the study population should not have dementia (either being cognitively intact, or having MCI). Studies investigating the role of NPS in dementia, and those dealing with specific NPS at preclinical/prodromal AD not defined as MBI (for instance, depression and anxiety), as well as in vitro, animal studies, reviews or other types of articles were excluded. No other exclusion criteria were set, including restrictions in the scales used for the MBI assessment, or methods used for the evaluation of amyloid or tau pathology (blood, cerebrospinal fluid, neuroimaging, and postmortem neuropathological studies). To explore the potential underlying mechanisms of MBI focusing on amyloid and tau pathology, as well as potential relative therapeutic implications, we additionally incorporated clinical, and animal studies or reviews exploring the role of amyloid or tau pathology in specific NPS—not defined as MBI—in non-demented individuals, such as depression, anxiety, and apathy, as well as NPS in dementia. We used Boolean operators (AND, OR, NOT) to combine our keywords in appropriate ways that align with our research aims, in order to ensure a systematic and comprehensive search of relevant literature. Abstracts were screened, relative articles were read in full-form, and data were extracted from included studies (study type, study population, MBI assessment, amyloid and/or tau pathology assessment, and main findings focusing on the role of amyloid or tau pathology in MBI). The bibliography of each relevant article was also screened for additional studies. Finally, 11 studies were included (Table 1). A narrative synthesis was performed, divided into the amyloid and tau pathology, as well as related pro-posed underlying mechanisms. Subdivisions were based on the type of biomarkers investigated (PET, CSF, blood, neuropathological samples) and type of study (cross-sectional, longitudinal).

## 3. Mild Behavioral Impairment (MBI): Definition, Clinical Features, Assessment Methods and Epidemiology

In order to operationalize the MBI syndrome and promote research into its relationship with cognitive decline, the International Society to Advance Alzheimer’s Research and Treatment (ISTAART) NPS Professional Interest Area—a subgroup of the Alzheimer’s Association (AA)—has published the ISTAART-AA MBI criteria in 2016 [19,25]. According to this definition, MBI can be considered as the complementary behavioral axis of preclinical dementia, in accordance with the traditional cognitive axis manifested as subjective cognitive decline (SCD) or MCI. These two axes can co-exist, and the presence of NPS in patients with MCI may further increase the risk of conversion to dementia [26]. MBI constitutes the development of sustained NPS in non-demented individuals in later life (>50 years of age), not attributed to a primary psychiatric disorder (major depression, general anxiety disorder, etc.) or other medical condition [19,25]. NPS should be present for at least six months, and constitute a noticeable change from the individual’s personality or usual behavior within the following five domains: (a) affective dysregulation (depressive symptoms, dysphoria, anxiety, euphoria), (b) decreased motivation and drive (apathy, indifference, aspontaneity), (c) impulse dyscontrol (irritability, disinhibition, agitation, aggression, obsessiveness, gambling, shoplifting), (d) social inappropriateness (loss of empathy, insight, or tact), and(e) abnormal perception or thought content (hallucinations, delusions) [19,25]. The criterion of 6 months aims to increase specificity, and specifically the probability that NPS are a manifestation of an underlying neurodegenerative disease and not caused by life events. The behavioral impairment should be severe enough to at least minimally affect functionality in social interactions, interpersonal relationships or work performance [19,25]. However, the individual should be functionally independent in activities of daily life, needing no or minimal assistance [25].

According to the National Institute of Aging-Alzheimer’s Association (NIA-AA) Framework, preclinical AD is represented by stages 1 and 2, and prodromal AD by stage 3. In stage 1, the individual is asymptomatic with objectively normal cognitive performance, in stage 2, subjective concerns and/or subtle cognitive deficits may exist, while in stage 3, there is objective cognitive impairment with functional independence. Even though the neurocognitive axis is the core characteristic in stages 2 and 3, MBI can co-exist or even be the main clinical feature [27].

The MBI-Checklist (MBI-C) has been constructed with the specific aim to standardize the assessment of MBI, serving as a case ascertainment tool for the detection and monitoring of NPS in pre-dementia populations [25]. MBI-C is composed of 34 questions categorized into the 5 MBI domains, and it can be completed by a close informant, clinician or the patient [25]. Since the development of the MBI-C is quite new, many studies have used other scales to assess MBI, with the Neuropsychiatric Inventory (NPI) or NPI Questionnaire (NPI-Q) being the most frequently employed. An algorithm for the transformation of the NPI or NPI-Q scores has been published, in order to approximate the characterization of MBI, with a change in time frame to 6 months in order to meet MBI criteria [28]. Based on this transformation, MBI is usually defined as having a score of >0 in NPI in at least one of the five corresponding MBI domains. The use of NPI or NPI-Q instead of MBI-C may provide less specificity, because of its shorter time reference range (1-month versus 6-months, respectively), resulting in the potential inclusion of individuals with transient behavioral alterations due to stressful life events or other reactive conditions. Because of this limitation, some studies have used the NPI or NPI-Q at two consecutive visits to define MBI, in case MBI-C assessments are not available [29]. On the other hand, given the fact that NPI and NPI-Q have been developed for patients with dementia, they may not be able to capture the full spectrum of mild NPS symptoms in the context of preclinical and prodromal AD, which may result in lower sensitivity [9]. For these reasons, the use of MBI-C is considered to be the optimal approach in assessing MBI.

In primary care settings, the prevalence of MBI has been found to be 14.2% and 5.8% among individuals with MCI (using the cut-off of 6.5) and SCD (using the cut-off of 8.5), respectively, as assessed by the MBI-C [30,31]. With the use of NPI instead of MBI-C at community settings, MBI prevalence is found to be higher in populations with MCI and SCD (83.5% and 76.5%, respectively) [28]. However, the prevalence and optimal cut-off points for individuals without cognitive complaints and normal cognitive function remain less clear. A large longitudinal study has shown that the presence of MBI, as evaluated by MBI-C, was correlated with worse cognitive function in cognitively intact individuals at baseline, and greater cognitive decline in 1 year, in the domains of attentional intensity, attentional fluctuation, sustained attention, and working memory [32].

## 4. The Role of Amyloid and Tau Pathology in the Neuropsychiatric Symptoms in Alzheimer’s Disease Dementia

Aβ is a 4 kDa peptide derived from the sequential proteolytic cleavage of amyloid precursor protein (APP) [33]. APP is a highly conserved transmembrane glycoprotein, consisting of three regions: the intracellular domain, the domain within the membrane, and the extracellular domain [34]. APP is highly concentrated in synaptic regions, and although its exact functional role remains uncertain, it is considered to be implicated in synaptic plasticity, neuronal survival, and intracellular transport [35]. Under normal conditions, APP is cleaved by alpha-secretase, to produce the membrane-tethered C-terminal C83 fragment, and the extracellularly released, and relatively short, N-terminal soluble APP alpha (sAPPα) fragment (non-amyloidogenic pathway) [33]. Alternatively, APP is cleaved by beta-secretase (also known as β-APP-cleaving enzyme-1 or BACE1) to produce the C-terminal membrane-tethered C99 fragment, and the extracellularly released, longer, N-terminal soluble APP beta (sAPPβ) fragment (amyloidogenic pathway) [33]. Although in the non-amyloidogenic pathway, gamma-secretase cleaves C83 to generate the APP intracellular domain (AICD) and P3, in the amyloidogenic pathway, gamma-secretase cleaves C99 to generate AICD and the extracellularly released Aβ [33].

An imbalance between Aβ formation and clearance can lead to amyloid pathology in the brain. The dysregulation of Aβ metabolism results in misfolding, the formation and the extracellular accumulation of hydrophobic, insoluble Aβ aggregates, including oligomers, fibrils, and senile plaques, which constitute the neuropathological hallmarks of AD. Aβ40 and Aβ42 are the two main types of amyloid with a central role in the formation of senile plaques and amyloid-induced neurodegeneration [36]. Aβ42 is insoluble, more neurotoxic and more prone to aggregation compared to Aβ40 [36]. Several mechanisms are implicated in the Aβ-related neurotoxicity, including autophagy and lysosomal impairment, proteasome dysfunction, oxidative damage, excessive neuroinflammation, mitochondrial impairment, dysregulated calcium homeostasis, abnormal lipid metabolism, and synaptic dysfunction [37]. In addition, Aβ can contribute to the aberrant hyperphosphorylation of tau, thereby enhancing the progression of tau pathology and promoting the development of amyloid plaques [38]. Several studies have shown that NPS might be related to amyloid pathology across the clinical spectrum of AD [39,40]. In particular, in a study including patients with MCI and AD dementia, NPS were associated with CSF AD biomarkers; affective symptoms, including depression and anxiety, were linked to lower levels of Aβ42, and apathy with an increased t-tau/Aβ42 ratio [39]. Apathy has also been correlated with higher amyloid deposition in the prefrontal cortex in patients with MCI and AD dementia [41], as well as lower Aβ42 levels in the CSF of patients with early-stage AD [42].

Tau is the major microtubule-associated protein (MAP) of mature neurons, mainly acting by stabilizing the microtubule network, which plays a crucial role in the maintenance of cell structure and intracellular transport [43]. Tau is a phosphoprotein, and its activity depends on its phosphorylation status [43]. Tau protein has 6 isoforms, depending on the 0, 1 or 2 sequence inserts in its N-terminus and the inclusion/exclusion of the microtubule-binding potential repeat domains (MTBD) [44]. According to the predominant tau isoforms in the intracellular inclusions, tauopathies are classified as 3R-tauopathies (primarily consisting of tau protein with 3 MTBDs), 4R-tauopathies (primarily consisting of tau protein with 4 MTBDs), and those with 3R and 4R tau protein in an equal ratio (3R:4R tauopathies) [44]. Under normal conditions, as well as in AD, tau exists in a 3R:4R equal ratio [44]. Tau can undergo post-translational modifications, such as phosphorylation at threonine and serine sites, which alters its ability to bind to microtubules [44]. In AD, tau consists of tightly paired helical filaments, whereas in other diseases, tau may form straight or twisted ribbon filaments [44]. In AD, tau undergoes abnormal hyperphosphorylation, leading toits reduced neurobiological activity and the formation of tau aggregates into insoluble NFTs [43]. The intracellular accumulation of NFTs impairs neuronal structure and function, finally resulting in neuronal death [43]. Although amyloid pathology is classically considered the initial process of AD-related neurodegeneration, postmortem evidence supports that tau pathology may be more directly correlated with global cognitive function, dementia clinical stage, functional status and NPS [45]. The spreading of tau pathology in the brain follows a pattern (Braak stages) that has been closely associated with the clinical progression of cognitive decline. Tau accumulation is rarely detected in individuals with normal cognition [46], and abnormalities in tau PET are generally observed in the context of existing amyloid deposition [46,47]. In this context, amyloid pathology has been suggested to be necessary for increased 3R/4R tau accumulation [46].

A growing number of studies support the role of tau pathology in NPS in AD. In particular, there is some evidence that NPS, such as depression, aberrant motor behavior and apathy, might be related primarily to tau, rather than amyloid pathology in AD [48,49]. In regard to specific NPS, anxiety has been associated with higher levels of p-tau and t-tau in the CSF inpatients across the spectrum of AD [40], and apathy has been related to CSF p-tau181 levels in patients with mild AD [50]. A postmortem study has shown increased levels of p-tau within neurons of the brains of AD patients with psychosis [51]. On the other hand, there is also evidence demonstrating no significant relationships between tau accumulation and global severity of NPS [52], as well as specific symptoms, including depression, anxiety, agitation, psychosis and irritability [52,53].

## 5. Clinical Evidence on the Potential Role of Amyloid Pathology in MBI

Based on the above evidence, it seems that both amyloid and tau pathologies, as well as an interconnection between them seem to be linked with NPS in AD, suggesting their potential role particularly in preclinical and prodromal phases, in terms of MBI. In this context, studies using amyloid PET have shown that MBI is associated with higher global and striatal amyloid burden, both cross-sectionally and longitudinally (Table 1) [54,55]. Cross-sectionally, Lussier and colleagues have demonstrated that MBI-C total scores were moderately correlated with greater amyloid accumulation in PET in non-demented individuals, in both cortical and subcortical regions, including the left frontal and posterior cingulate cortex, as well as the left thalamus and caudate nucleus, respectively [55]. Importantly, the brain regions with the strongest correlation corresponded to areas displaying initial sites of AD-related amyloidosis, particularly neocortex and striatum [56,57]. However, in another study by Chan and colleagues no significant association was observed between MBI and amyloid pathology assessed by PET [58]. The different sample sizes, and the different assessments used for the evaluation of MBI might explain these contradictory results. In agreement with this evidence, a higher burden of NPS was correlated with higher amyloid deposition in amyloid PET, in a study among cognitively normal individuals at a high risk of AD (due to a positive family history of sporadic AD) [52]. Longitudinally, more severe NPS in the context of MBI have been correlated with more rapid increase in amyloid burden in PET in individuals with normal cognition and MCI, as well as a higher risk of cognitive decline [54]. In agreement with this evidence, amyloid accumulation has been associated with an increase in depressive, anxiety and apathy symptoms over time in non-demented individuals [59,60].

Interestingly, another study by Matsuoka and colleagues aiming to identify differences in NPS between preclinical/prodromal AD and prodromal PDD/DLB demonstrated that MBI was related to both amyloid pathology assessed by PET and putative Lewy body pathology assessed by dopamine transporter single photon emission computed tomography (DAT-SPECT) [64]. More specifically, the score of the MBI domain of psychosis was higher in the group with concurrently abnormal amyloid PET and DAT-SPECT, compared to the group with normal cognition, amyloid PET and DAT-SPECT [64]. In addition, the scores of MBI as a global measure and the domain of impulse dyscontrol were higher in the group with MCI unlikely due to AD and normal DAT-SPECT compared to the group with normal cognition, amyloid PET and DAT-SPECT [64]. Although DAT-SPECT is a marker of an underlying presynaptic dopaminergic neurodegeneration and not a direct indicator of underlying Lewy body pathology, these findings suggest that psychotic features in the context of MBI in non-demented individuals might be more common when these co-pathologies coexist.

Concerning the assessment of amyloid deposition via CSF biomarkers, a recent study by Ismail and colleagues based on two different cohorts demonstrated that MBI was associated with amyloid pathology at a both cross-sectional and longitudinal level [9]. In particular, MBI was cross-sectionally related to lower Aβ42 levels in both ADNI and MEMENTO cohorts, but with a lower Aβ42/40 ratio only in ADNI [9]. This difference was attributed by the researchers to the less strict inclusion criteria in the MEMENTO cohort, possibly resulting in higher likelihood of inclusion of participants with factors linked to lower Aβ40 levels, such as nicotine use or vascular burden [9]. On the contrary, NPS-not-MBI was only related to the reduced Aβ42/40 ratio in the ADNI cohort [9], highlighting the importance of using MBI criteria for defining NPS in the context of increased risk of neurodegeneration. In agreement with this evidence, anxiety, agitation and irritability have been linked to lower Aβ42 levels in the CSF in individuals with MCI, and apathy has been associated with a lower CSFF Aβ42/total tau ratio in non-demented individuals [66].

Blood-based biomarkers represent a minimally invasive method for the early detection of individuals at risk for AD. In particular, the plasma Ab42/Ab40 ratio has been shown to be a good predictor of brain amyloid deposition in older cognitively normal individuals [67], highlighting its scalable usefulness in assessing the underlying amyloid pathology. In this context, in the study by Miao and colleagues, total MBI score has been cross-sectionally correlated with a lower plasma Ab42/Ab40 ratio in non-demented participants [29]. Among the different MBI domains, significant associations were found only for affective dysregulation [29]. Notably, the plasma Ab42/Ab40 ratio did not significantly differ between groups with MCI and normal cognition in this study, further supporting the hypothesis that MBI might be an earlier and more sensitive clinical marker of amyloid neuropathology compared to MCI [29].

Regarding clinicopathological evidence, there is only one study by Ruthirakuhan and colleagues, based on longitudinal clinical evidence and postmortem examination of brain samples, which demonstrated that MBI was an important predictor of the progression to both the clinical and neuropathologically confirmed diagnosis of AD [20].

Concerning the link between MBI and cognitive decline, the study by Sun and colleagues revealed that the association between MBI and cognitive impairment was at least partially mediated by amyloid pathology [54]. Interestingly, this mediation effect was observed for both global cognition, as well as specific cognitive domains, including memory, language and executive function [54]. Depressive symptomatology has also been linked to worse global cognitive function and episodic memory only in cognitively normal individuals with both amyloid pathology and evidence of neurodegeneration [68]. Worsening depressive symptoms over time have also been longitudinally related to cognitive decline in cases with higher and not lower amyloid burden [69]. Although causality cannot be determined, these findings imply that amyloid pathology might at least partially mediate the association between MBI and cognitive decline. On the contrary, another study by Chan and colleagues in 2022 demonstrated that amyloid burden assessed by amyloid PET did not affect the relationship between MBI and cognitive decline [58].

The role of amyloid pathway-related genetic factors in MBI remains largely unexplored. In this regard, in the study by Sun and colleagues, the relationship between MBI and higher amyloid burden remained significant after adjusting for cognitive status (normal cognition vs. MCI), age, sex, education years and the presence of APOEe4 allele [54]. On the other hand, the presence of APOEe4 allele has been associated with greater anxiety symptoms in non-demented individuals with subcortical amyloidosis [70]. Furthermore, a cross-sectional study including 117 non-demented individuals carrying gene mutations related to the autosomal dominant-inherited AD (APP, PSEN1 and PSEN2), demonstrated that a greater burden of neuropsychiatric symptoms, as assessed by NPI-Q, was related to increased amyloid pathology in amyloid PET [52]. This evidence suggests that in cases of genetic predisposition to amyloid dysregulation, the presence of NPS in preclinical or prodromal dementia stages might reflect a more pronounced underlying amyloid pathology.

Collectively, MBI seems to be associated with amyloid pathology as evaluated by amyloid PET, CSF and blood-based biomarkers, as well as neuropathological postmortem examination. Although this relationship does not establish causality, it highlights the potential role of amyloid-related pathways in the development of MBI and its link with cognitive decline. However, additional pathophysiological mechanisms not related to AD pathology are probably involved. It is still unclear under which conditions amyloid-related mechanisms contribute the most to MBI and/or its progression to dementia, and which other factors might affect this relationship. This knowledge would be very important, since it would aid in the development of more effective preventive and therapeutic interventions.

## 6. Clinical Evidence on the Potential Role of Tau Pathology in MBI

In contrast to amyloid pathology, the results from clinical studies investigating the relationship between MBI and tau biomarkers are relatively less consistent. In particular, in a recent study by Johansson and colleagues among cognitively unimpaired individuals with amyloid positivity evaluated by CSF, MBI was associated with tau pathology evaluated by tau PET and CSF [63]. In particular, higher MBI-C scores were correlated with increased signal in the entorhinal cortex and hippocampus (Braak region I–II) in tau PET, as well as higher p-tau181 levels in the CSF [63]. However, MBI was not related to tau pathology in the other Braak regions III–IV (parahippocampal cortex, amygdala, fusiform cortex, inferior temporal cortex, and middle temporal cortex) and V-VI (widespread neocortical regions) [63]. Importantly, in combined statistical models incorporating both MBI-C scores and episodic memory performance, only MBI was associated with tau pathology [63]. These results suggest that MBI might be a potential early marker of AD-related tau pathology before and independently of cognitive decline. Concerning the different MBI domains, only affective dysregulation and impulse dyscontrol were linked to greater tau pathology in PET in the Braak regions I–II, as well as higher p-tau181 levels in the CSF [63].

On the contrary, although MBI was related to amyloid pathology, no significant relationships were detected for tau pathology in PET among non-demented individuals both cross-sectionally [41,54], and longitudinally [54]. However, in contrast to the study by Johansson and colleagues, these studies also included individuals without amyloid pathology, and the different methods used characterizing MBI might have contributed to the different results. For instance, the study by Sun and colleagues used NPI-Q in a single visit for defining MBI, possibly resulting in lower specificity [54].

With the aim to better clarify the role of tau pathology in MBI, another recent study by Naude and colleagues using amyloid and tau PET among non-demented individuals demonstrated that amyloid pathology status affected the relationship between MBI and tau pathology in the brain regions corresponding to the Braak I and III stages of AD [65]. In particular, in the group with amyloid pathology, MBI was related to tau uptake in Braak I and Braak III regions, whereas in the group without amyloid pathology, MBI was only negatively associated with tau uptake in Braak III [65]. The negative correlation between MBI and tau pathology in the case of absence of amyloid abnormality in this study suggests that other pathologies, such as vascular, TDP-43, non-helical tau, only 3R or 4R tauopathies (and not the paired helical filament form, or the combination of 3R and 4R tau forms observed in AD, detected with AV145Cby tau PET in this study) would underlie the pathophysiology of MBI [65]. Collectively, these findings suggest that AD-related, and not minor age-related tauopathy might be the main pathophysiological contributors to MBI [65]. Future studies investigating the potential underlying pathology of MBI in the case of amyloid negativity are required.

In agreement with these results, NPS including depression, anxiety and agitation evaluated by NPI were related with tau pathology in Braak I and II regions in a postmortem study [71]. Braak II region (hippocampal area) was not included in this study for technical reasons. However, considering the study by Johansson and colleagues mentioned above, in which MBI was also linked to Braak II region, it seems that MBI follows a typical progression of AD neuropathology [65].

In line with this evidence, depressive symptoms among cognitively normal individuals have been associated with increased tau pathology in the inferior temporal and entorhinal cortex, assessed by tau PET [72]. Furthermore, a study in cognitively normal individuals at a high risk of AD due to a positive family history of AD demonstrated that tau deposition evaluated in tau PET was related to a higher burden of NPS [40]. Importantly, the strongest relationship was observed in the tau signal in PET in the entorhinal cortex, further supporting the hypothesis that MBI, as an initial clinical feature, reflects the early stages of the temporal progression of tau pathology in AD.

Another clinical study in individuals with normal cognition, MCI and AD dementia aimed to investigate the relationship between the integrity of the locus coeruleus (LC)-norepinephrine system, AD stage and MBI, using tau and amyloid PET, as well as the neuromelanin-sensitive MRI technique [62]. In this study, LC signal loss was indicated to AD stage assessed by Braak stages and clinical severity [62]. While no significant associations were identified between LC signal and MBI-C score in cognitively intact participants, in the group of tau-positive individuals, a preserved signal in the LC was correlated with higher risk of MBI [62]. This association was shown to be driven primarily by the MBI domain of impulse dyscontrol [62]. These findings suggest that NPS in patients with MCI and dementia in AD are linked to preservation of LC and increased function of norepinephrine, possibly due to compensatory mechanisms related to norepinephrine generation, expression of receptors and alterations in axon terminals [62].

Concerning studies using only CSF, the study by Ismail and colleagues mentioned above indicated that that MBI was associated with tau pathology in the CSF at a both cross-sectional and longitudinal level in two different cohorts [9]. In particular, MBI was related to higher CSF p-tau and t-tau levels, as well as the p-tau/Aβ42 and t-tau/Aβ42 ratios [9]. In another study among cognitively intact individuals, although no significant relationships were detected between NPS and CSF p-tau181 at a cross-sectional level, increasing scores in NPI-Q were associated with CSF p-tau181 at one year in follow-up [73]. Consistent with this evidence, anxiety has been linked to higher t-tau levels in the CSF in individuals with MCI [74].

In accordance, plasma phosphorylated tau at threonine 181 (p-tau181) is a promising blood-based AD biomarker predicting progression to AD dementia with very good sensitivity [75,76,77]. In a recent study by Ghahremani and colleagues, MBI was both cross-sectionally and longitudinally associated with increasing plasma p-tau181 levels compared to no-NPS in non-demented older individuals, while no significant differences were found between NPS-not-MBI and no-NPS [61]. In this study, interaction analyses revealed that in the p-tau181-positive group, MBI was related with a higher risk of incident dementia compared to no-NPS, while no significant relationship was observed for NPS-not-MBI [61]. These findings highlight the specificity of MBI and the importance of MBI criteria for the pre-dementia phases.

As above mentioned, MBI was identified in the clinic-pathological study by Ruthirakuhan and colleagues as a predictor of progression to neuropathologically confirmed diagnosis of AD [20], further supporting its relationship with AD-related tauopathy.

Therefore, emerging evidence suggests that MBI is at least partially associated with amyloid and tau pathology in non-demented individuals, suggesting that both amyloid- and tau-related pathways are probably implicated in its development. The relatively stronger evidence regarding the association between MBI and amyloid compared to tau pathology further supports the prevailing assumption that MBI is an early clinical manifestation of the AD-related neurodegenerative process, when tau pathology might not have become evident [54]. In addition, it seems that in existing amyloid pathology, MBI is associated with the extent of tau pathology, corresponding to the Braak stages of the neuropathological progression of AD. Compared to amyloid deposition, tau pathology correlates better with cognitive impairment in AD [49], and NPS seem to also follow this pattern in the AD clinical spectrum including MBI. On the other hand, in the absence of amyloid pathology, MBI may be linked to other non-AD pathophysiological mechanisms, such as vascular, TDP-43, or non-AD tau pathologies, including non-helical tau, as well as only 3R or 4R tauopathies. Further longitudinal studies with large sample sizes, with long, regular follow-up of NPS symptoms with MBI-C, AD neuropathology and cognitive status including both neuropsychological testing and assessment of functionality, would help us clarify the complex temporal relationship between underlying neuropathologies and MBI.

## 7. Proposed Amyloid- and Tau-Related Underlying Pathophysiological Mechanisms of MBI

**Figure 1:**
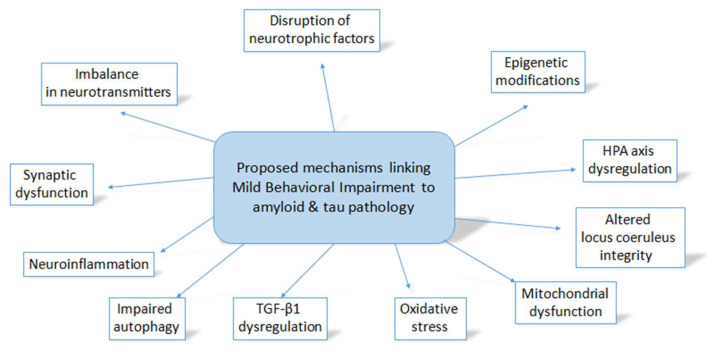
The potential role of amyloid and tau pathology in MBI.

Although the underlying pathophysiological mechanisms of MBI remain unexplored, the existing literature evidence from clinical and animal studies in NPS in AD provide insights into its potential amyloid- and tau-related neurobiology (Figure 1). Interestingly, mouse models of Aβ amyloidosis display behavioral alterations reflecting emotional dysregulation, before the appearance of obvious cognitive impairment, suggesting that NPS might precede the onset of cognitive decline in AD [78]. Subtle behavioral alterations, primarily in anxiety- and social-related domains, have been also observed early in mouse models of Aβ amyloidosis, before the prominent deposition of amyloid plaques [79]. These findings further support the role of amyloid pathology in the early development of NPS in AD, even before the onset of cognitive deficits.

### 7.1. The Hypothalamic–Pituitary–Adrenal (HPA) Axis

A growing body of evidence suggests that chronic psychological stress may increase the risk of AD [80]. The hypothalamic–pituitary–adrenal (HPA) axis constitutes the primary pathway of stress response, modulating cortisol production [80]. Higher cortisol levels and HPA axis dysregulation are commonly observed in patients with AD, and have been proposed to be critically involved in the pathophysiology of the disease [80]. In this context, it has been proposed that amyloid-induced neurotoxicity might contribute to the HPA axis dysfunction and elevate glucocorticoid levels, thereby resulting in affective dysregulation. APP has been demonstrated to be endogenously expressed in crucial hypothalamic, limbic, and midbrain nuclei involved in the modulation of the activity of the HPA axis [81]. In a study with Aβ(25-30)-induced rat models of AD, amyloid has been related with altered anxiety response, abnormal feedback response of the HPA axis, as well as higher levels of glucocorticoid and mineralocorticoid receptor in brain areas implicated in the HPA axis [82]. In another study with APP transgenic mouse models of AD, intraneuronal amyloid deposition has been shown to dysregulate the amygdala-dependent emotional responses, possibly by modulating the extracellular signal-regulated kinase (ERK)/mitogen-activated protein kinase (MAPK) signaling pathway [83]. Hence, it could be speculated that the amyloid pathology-mediated HPA axis disruption potentially via the modulation of the ERK/MAPK pathway might at least partially contribute to the pathophysiology of MBI, and particularly the domain of affective dysregulation.

### 7.2. Dysregulation of Neurotrophic Factors

Depression and AD share some common neurobiological mechanisms, including the dysregulation of neurotrophic factors, such as the brain-derived neurotrophic factor (BDNF) [84]. In major depressive disorder, BDNF levels have been correlated with symptom severity, and antidepressant treatment may increase its levels [85]. BDNF has been demonstrated to be reduced at a both mRNA and protein level in the hippocampal region and neocortex of patients with AD [86]. BDNF depletion has been associated with tau hyperphosphorylation, Aβ deposition, neuronal apoptosis and neuroinflammation in AD [87]. The efficacy of paroxetin, a selective serotonin reuptake inhibitor (SSRI), in AD-related depression has been shown to depend on the BDNF G196A gene polymorphisms, since A allele carriers display better therapeutic response [88]. BDNF can dephosphorylate tau, thereby possibly affecting its binding ability with microtubules, via the activation of TrkB and a PI3-kinase/Akt-dependent mechanism [89]. In addition, oligomeric forms of Aβ can inhibit BDNF transcription in vitro [90]. These results suggest that the BDNF pathway might be implicated in AD-related depression and the affective dysregulation domain of MBI, potentially via amyloid- and tau-related pathways.

### 7.3. Neuroinflammation

Neuroinflammation represents another interconnecting link between AD and depression, and amyloid pathology seems to be also implicated in this relationship. The kynurenine (Kyn) pathway is one of the potential mechanisms linking AD-related depression with neuroinflammation, amyloid and tau pathology. In response to inflammation, pro-inflammatory cytokines can upregulate indoleamine 2,3-dioxygenase (IDO), resulting in the metabolism of tryptophan (Trp)—the amino acid precursor of serotonin—to kynurenine. Higher expression of IDO has been observed in the cortex and hippocampus of patients with AD, being also associated with Aβ burden [91]. AD patients also display a higher Kyn/Trp ratio, which reflects a higher IDO activity and increased tryptophan degradation [92]. Interestingly, the Kyn pathway has been implicated in cognitive impairment, negative affect, as well as amyloid and tau pathology in AD [93]. In particular, amyloid pathology affected the relationship between Kyn/serotonin and the scores in negative affect, and the complement system seemed to completely account for this relationship in the presence of amyloid pathology [93].

### 7.4. Dysregulation of the Transforming-Growth-Factor-β1 (TGF-β1) Signaling Pathway

Transforming-growth-factor-β1 (TGF-β1) is an anti-inflammatory cytokine with a potential beneficial role in depression and Aβ-induced neurodegeneration. AD patients also demonstrate reduced levels of TGFβ-1 in their superior temporal gyrus, which is correlated with the degree of tau accumulation [94]. Animal studies have indicated that impaired TGF-β1 signaling may be implicated in both amyloid and tau pathology in AD [84]. Cell cycle regulation, GSK-3β inhibition, suppression of Aβ-induced tau hyperphosphorylation, and its anti-inflammatory effects are the main proposed underlying neuroprotective mechanisms of TGF-β1 [84]. Patients with major depression display lower plasma levels of TGF-β1, which are also associated with symptom severity and treatment response [84]. Fluoxetine, can also promote TGF-β1 astrocytic release, thereby playing a neuroprotective role in animal models of AD [95]. Hence, TGF-β1 activity might be also crucially involved in the amyloid- and tau-related pathophysiology of affective dysregulation in the context of MBI, and further research is needed towards this direction.

### 7.5. Epigenetic Modifications

Epigenetic regulation, including DNA methylation, histone modifications and micro-RNAs (miRs) have also been implicated in NPS in the spectrum of AD. Plasma levels of miR-451a, among other miRs, have been correlated with AD and depression [96]. A recent study has shown that levels of miR-451a in the CSF negatively correlated with cognitive deficits and depressive symptoms in patients with AD [97]. Mechanistically, miR-451a overexpression of miR-451a in the medial prefrontal cortex of transgenic mouse models of AD could inhibit memory impairment, depression-like behavior, neuroinflammation and Aβ burden [97]. miR-451a could also downregulate the expression of β-secretase 1 (BACE1) in neuronal cells by suppressing the Toll-like receptor 4 (TLR-4)/NF-κB pathway, as well as inhibit the activation of microglia by suppressing NOD-like receptor protein 3 [97]. Furthermore, miR-455-3p is increased in the brain of patients with AD, while its overexpression has been shown to reduce the expression of TAU, APP and BACE1 in neuroblastoma cells [98]. miR-455 null mice display cognitive impairment, increased anxiety, accompanied by higher tau, APP and BACE1 levels in the hippocampus of the animals [98].

### 7.6. Synaptic Dysfunction and Neurotransmitter Imbalance

Importantly, synaptic dysfunction and neurotransmitter imbalances, and primarily acetylcholine, dopamine, noradrenaline, serotonin, glutamate, and gamma-aminobutyric acid (GABA) are critically involved in the pathophysiology of NPS in AD, highlighting their potential role in MBI possibly via the amyloid- and tau-related pathways [99]. Aβ deposition impairs synaptic vesicle cycling, the release of neurotransmitters in the synaptic cleft, thereby affecting synaptic homeostasis [100]. In this regard, the development of apathy, which has been correlated with impaired dopaminergic neurotransmission during aging, has been at least partially associated with the amyloid-mediated inhibitory effects on the release of dopamine [101]. Moreover, Aβ can suppress the release of glycine and GABA, possibly contributing to the development of psychotic manifestations [100].

### 7.7. Locus Coeruleus (LC)-Norepinephrine System Dysregulation

The locus coeruleus (LC) is the primary brain area of norepinephrine production, and one of the initial regions of tau accumulation in AD even years before cognitive decline [62]. However, despite the early tau pathology, postmortem evidence suggests that the neurodegenerative process in the LC has been shown to be relatively slow [102]. Alterations in the LC-norepinephrine system have been linked to both cognitive and behavioral symptoms in AD [103]. The pathophysiological mechanisms of the LC impairment in AD are rather complex, since compensatory alterations might lead to hyperactivated LC neurons in response to neurodegeneration [62]. Higher norepinephrine levels are observed in the CSF of patients with AD [104]. NPS in AD, including aggressiveness, agitation and psychotic manifestations have been correlated with preserved or higher norepinephrine-system activity [62]. As described above, the study by Cassidy and colleagues indicated that in tau-positive individuals, a preserved LC-norepinephrine system was associated with higher MBI-C scores correlated with higher risk of MBI, and this association was primarily driven primarily by impulse dyscontrol [62]. Based on this evidence, it has been proposed that cortical tau accumulation might modulate in a top-down mechanism the responses to arousing or stressful conditions in the case of an hyperactivated or intact LC, resulting in impulse control dysregulation, aggressiveness, agitation or other related NPS [62]. Dysregulation of LC-norepinephrine system and degeneration of LC neurons has been associated with enhanced neuroinflammatory responses, higher levels of tau accumulation, dysfunction of the blood–brain barrier (BBB), and impaired Aβ degradation. NPS in individuals in the AD spectrum have been linked to altered norepinephrine metabolism and p-tau [105]. Collectively, it can be hypothesized that tau-mediated LC-norepinephrine system dysregulation might contribute to MBI development in the context of AD, and particularly the domain of impulse dyscontrol.

### 7.8. Neuroanatomical Correlates of MBI

Regarding the neuroanatomical correlates of MBI, the association between MBI and tau pathology in the hippocampus and entorhinal cortex suggests the potential role of these regions in the early development of NPS in preclinical AD. A pronounced interconnection is observed between the hippocampal region and the amygdala, a brain area highly involved in emotional regulation. In addition, the amygdala has been proposed to interact with the hippocampus during the formation of long-term memories, and both regions are parts of several brain circuits implicated in cognitive and emotional processing [106,107]. Another study in individuals with normal cognition, MCI and AD dementia has demonstrated that the severity of NPS was related to tau pathology in tau PET, and this correlation was greater in the superior frontal, medial occipital and temporal lobes, as well as the parietal association area, which are early affected by AD neuropathology and constitute components of neurobehavioral brain circuits [49]. Interestingly, in this study, specific NPS were linked to specific regions of tau pathology, implying that the topographical pattern of tau accumulation might be related to the heterogeneity of NPS in the spectrum of AD [49]. For instance, some of the detected relationships involved agitation with paralimbic regions, anxiety with superior medial frontal gyrus, depressive symptoms with the lateral superior temporal gyrus, and irritability with the frontal pole [49]. These findings pave the way for the further investigation of the topographical pattern of tau pathology in the different MBI domains.

## 8. Therapeutic Implications

Given the emerging literature evidence supporting the link between MBI and dementia, as well as AD biomarkers, it can be speculated that therapeutic interventions targeting mild NPS in individuals with normal cognition or MCI would be beneficial, especially in mitigating the risk of subsequent cognitive decline. However, it is still unknown if MBI is a reversible condition, and future research is needed towards this direction. Based on the available clinical, in vivo and in vitro evidence, it can be speculated that selective serotonin reuptake inhibitors (SSRIs), norepinephrine blockade agents, cholinesterase inhibitors, several other pharmacological compounds including PDE4 inhibitors, as well as non-pharmacological approaches such as physical exercise might be helpful in addressing MBI symptoms and dementia risk, at least partially via amyloid- and tau-related mechanisms.

### 8.1. Selective Serotonin Reuptake Inhibitors (SSRIs)

Antidepressant therapies and especially SSRIs have been shown to promote neurogenesis, inhibit neuroinflammation, as well as decrease amyloid and tau pathology [108]. In APP/PSEN1 transgenic mouse models of AD, citalopram could also reduce the formation of new amyloid plaques and inhibit the growth of the existing ones in the brain [109]. The neuroprotective effects of citalopram, a SSRI, have also been investigated in vitro, in serotonergic, medullary dorsal raphe neurons transfected with mutant APP and tau [108]. In particular, treatment with citalopram was associated with increased neuronal survival, lower levels of p-tau, upregulation of genes expressing synaptic proteins, as well as improved mitochondrial respiration and morphology [108]. Furthermore, citalopram treatment has been related to improved autophagy, mitochondrial function and increased expression synaptic genes in another study using immortalized mouse primary hippocampal cells expressing mutant APP [110]. Interestingly, pimavanserin, a 5HT2A receptor inverse agonist that has been approved for the treatment of Parkinson’s disease-related psychosis, has been shown to improve cognitive function and inhibit the production of Aβ and amyloid pathology in transgenic mouse models of AD [111]. Therefore, SSRIs and pimavanserin might exert neuroprotective effects in AD-related neurodegeneration, at least partially by regulating mitochondrial function, autophagy, synaptic function, as well as the amyloid- and tau-related pathways, paving the way for future research in MBI and related cognitive decline.

At a clinical level, a decrease in CSF amyloid levels has been observed after a single dose of the SSRI citalopram in healthy individuals, implying a direct effect of SSRIs on the amyloid-related pathways [109]. The use of SSRIs may enhance cognitive function in patients with MCI [112]. Furthermore, the use of SSRIs by patients with MCI and depression for at least 4 years has been associated with a delay of progression to AD dementia by 3 years [113]. Hence, SSRIs for addressing MBI and particularly affective dysregulation in patients with MCI might constitute a promising novel therapeutic strategy, necessitating further research.

### 8.2. Noradrenergic and Norepinephrine Blocking Medications

Noradrenergic and norepinephrine blocking agents, such as prazosin, have been demonstrated to be effective against apathy, as well as agitation and aggressiveness in AD [114,115]. Methylphenidate, a noradrenergic and dopaminergic agent, has been investigated in several clinical trials for apathy in AD with some promising results [115]. Importantly, given the relationship between LC integrity and MBI in the study by Cassidy and colleagues, neuromelanin-sensitive MRI might serve as a potential biomarker indicating individuals with a higher likelihood of treatment response [62].

### 8.3. Cholinesterase Inhibitors

Cholinesterase inhibitors have been demonstrated to improve NPS in addition to cognitive symptoms in AD, highlighting their promising potential in MBI as well. In particular, donepezil treatment in patients with mild-to-moderate AD has been associated with improved global NPS assessed by NPI [116], and the greatest effects were observed for mood disturbances and delusions in another study including patients with AD and severe NPS [117]. It has been also demonstrated that donepezil may exert therapeutic benefits in behavioral symptoms especially in patients with severe AD [118], implying that its use might not be ideal for patients with mild NPS in preclinical/prodromal stages of AD in the context of MBI.

### 8.4. Other Pharmacological Compounds

Moreover, pharmacological compounds have been shown to exert some benefits in NPS in AD animal models potentially viathe amyloid-related pathways, implying that they might be helpful for MBI. In particular, it has been demonstrated that glibenclamide, an ATP-sensitive potassium-channel inhibitor, has been associated with decreased levels of anxiety- and depressive-like behavior in rat models of AD treated with Aβ25-35, possibly by restoring the activity of the HPA axis [119]. In addition, apelin-13 has shown anxiolytic effects in rat models of AD treated with Aβ25-35, possibly via upregulating the expression of the glucocorticoid receptor (GR) and downregulating those ofFK506 binding protein 51 (FKBP5), which play a pivotal role in the regulation of the HPA axis [120]. Furthermore, minocycline, a tetracycline antibiotic has attracted increasing attention for the treatment of neuropsychiatric disorders; in rat models of AD treated with Aβ1-42, minocycline administration has been associated with decreased depression-like behavior, accompanied by lower levels of the pro-inflammatory cytokines interleukin(IL)-1β and tumor necrosis factor (TNF)-α in the hippocampus of the animals [121]. Melatonin has been also shown to reverse cognitive decline and anxiety- and apathy-like behaviors in transgenic mouse models of AD, possibly by downregulating the nuclear factor kappa B (NF-κB) pathway, enhancing the activity of proteasome, as well as upregulating the SIRT1 pathway that promotes neuroprotection and longevity [122]. Nattokinase has been demonstrated to improve the Aβ-induced cognitive impairment, as well as anxiety-, depression-like behavior in mouse models of AD, possibly via the downregulation of the pro-inflammatory cytokines IL-6 and TNF-α, as well as the upregulation BDNF and the anti-inflammatory cytokine Il-10 [123].These findings suggest that these agents might hold a promising therapeutic potential in MBI and especially the domains of affective dysregulation and apathy in AD, mainly through their effects on the HPA axis regulation and neuroinflammatory responses.

Phosphodiesterase-4 (PDE4), a member of the PDE superfamily, is critically implicated in the modulation of cyclic AMP (cAMP) levels. In turn, cAMP acting as a second messenger, can activate protein kinase A, leading to the subsequent phosphorylation of the cAMP-response element binding (CREB) protein. The CREB/BDNF pathway plays an important role in several neuronal functions, including synaptic plasticity, release of neurotransmitters, neurogenesis and neuroprotection. Aβ has been demonstrated to interact and activate PDE4D5 in neurons, leading to lower cAMP availability [124]. Interestingly, rolipram, a PDE4 inhibitor, has been shown to improve cognitive function, as well as depression- and anxiety-like behavior in transgenic mouse models of AD, possibly by inhibiting apoptosis, amyloid pathology, tau phosphorylation and neuroinflammation [125]. Potential underlying pathways involved the stimulation of cAMP/PKA/26S proteasome and cAMP/exchange protein activated by cAMP (EPAC)/ERK pathways [125]. Rolipram was also related to reversed Aβ-induced cognitive deficits, possibly by modulating the HPA axis, the cAMP/cAMP response element-binding protein (CREB)/BDNF pathway, as well as levels of corticosterone, glucocorticoid receptors, and corticotropin-releasing factor [126].Another study in transgenic mouse models of AD has shown that roflumilast, another PDE4 inhibitor, was linked to improved cognitive function and depression-like behavior, possibly via PDE4B/PDE4D-mediated cAMP/CREB/BDNF signaling [127].Roflumilast has also been related to improved depression-like behavior in rat models of depression, potentially via anti-inflammatory, anti-oxidant, and neuroprotective mechanisms, including upregulation of BDNF and superoxide dismutase (SOD), as well as downregulation of pro-inflammatory cytokines such as IL-6 [128]. Given the dual effects of PDE4 inhibitors on both cognitive impairment and depression in AD, the neuroprotective potential of PDE4 inhibitors should be also explored in the context of MBI.

### 8.5. Combined Pharmacological Approaches

Combined pharmacological approaches, including antidepressant and acetylcholinesterase inhibitors might be also considered in MBI, especially in cases with MCI. In this regard, a recent study in mixed animal models of AD dementia and depression, demonstrated that the combined administration of fluoxetine and galantamine, as well as fluoxetine and donepezil could reverse both cognitive impairment and depressive-like behaviors, such as anhedonia [129]. Furthermore, fluoxetine and donepezil treatment were associated with lower hippocampal IL-10 levels in the animals [129]. Hence, it can be speculated that such combined therapies might exert beneficial effects on MBI in the context of AD, especially regarding affective dysregulation.

### 8.6. Non-Pharmacological Interventions

Non-pharmacological interventions, such as physical exercise, might be promising for ameliorating MBI symptoms and reduce the risk of dementia via amyloid- and/or tau-related mechanisms. In this context, long-term physical exercise pre-treatment has been linked to improved anxiety- and depression-like behavior in transgenic rat models of AD [130]. These alterations were accompanied by decreased tau hyperphosphorylation, lower levels of amyloid deposition, preserved synaptic density, improved mitochondrial function, as well as suppressed neuroinflammation, oxidative stress and neuronal damage [130]. In another study, a treadmill exercise could exert prophylactic effects on the Aβ-induced depressive-like behavior in mice, with inhibition of gut dysfunction being possibly implicated as an underlying mechanism [131]. Furthermore, swimming exercise has been related to improved anxiety- and depression-like behavioral and cognitive deficits in mouse models of AD, potentially by increasing BDNF, as well as reducing the levels of glutamate and pro-inflammatory cytokines such as TNF-α in the hippocampus of the animals [132]. Physical exercise has also been associated with improved apathy-like behavioral symptoms possibly by regulating the CREB/BDNF pathway in post-menopausal mouse models of AD [133]. Beyond physical exercise, the role of other non-pharmacological approaches, such as cognitive training, should be also investigated in the context of MBI.

## 9. Discussion and Future Directions

Based on the abovementioned evidence, several interesting conclusions and suggestions can be made. Firstly, the relationship between MBI and amyloid pathology further supports the characterization of MBI as a potential early clinical marker of AD neuropathology. Secondly, the correlation between MBI and tau pathology especially in existing amyloid deposition seems to correspond to the Braak stages of AD progression, suggesting that MBI might be also an indicator of an accelerated disease course. In the absence of amyloid deposition, MBI may be related to other non-AD pathologies, such as vascular, Lewy body or non-AD types of tau deposition, which should be explored in future studies. Thirdly, it seems that the use of MBI criteria including the important factor of persistence is crucial in reducing the noise and increasing specificity, since it lowers the likelihood of including individuals with transient or reactive NPS. Finally, based on the relationship between MBI and both amyloid and tau pathologies, several underlying pathophysiological mechanisms can be proposed, which need to be further investigated at a both clinical and preclinical level. Dysregulation of the HPA axis, disruption of neurotrophic factors, such as the BDNF, abnormal neuroinflammatory responses including the Kyn pathway, oxidative stress, impaired autophagy, mitochondrial dysfunction, dysregulation of TGF-β1, epigenetic alterations including miR-451a and miR-455-3p, synaptic dysfunction, imbalance in neurotransmitters including acetylcholine, dopamine, serotonin, GABA and norepinephrine, as well as altered LC integrity are some of the potential mechanisms connecting MBI with amyloid and tau pathology. The elucidation of the underlying neurobiology of MBI would facilitate the design and efficacy of relative clinical trials, especially towards the amyloid- or tau-related pathways.

A growing body of evidence supports the crucial implication of neurotoxic soluble amyloid beta oligomers in the early stages of AD, as the potential upstream pathogenic drivers. Neurotoxic soluble amyloid beta is involved in tau hyperphosphorylation, synaptic dysfunction, and neuronal loss [134]. Soluble amyloid beta subtypes range from dimers up to dodecamers, as well as larger amyloid beta protofibrils, with each of them displaying a distinct pattern of neurotoxicity. Soluble Aβ oligomers can further aggregate into insoluble beta sheets, thereby forming amyloid fibrils and plaques, which can be identified in AD patients with amyloid PET [134]. Insoluble amyloid beta aggregates into plaques can be found extracellularly, both in the brain parenchyma and blood vessels (amyloid angiopathy). Although amyloid plaques are one of the main hallmarks of AD, their exact role has not been elucidated; they considered to represent a later stage of AD, being associated with neuroinflammation, and neuronal network disruption. On the other hand, amyloid beta plaques may act as a compensatory mechanism. The identification of the amounts of soluble beta amyloid is challenging, and it can be classically performed in the CSF or postmortem tissue lysates, each with inherent limitations [135]. Although CSF represents a relatively accessible technique, it is invasive, and soluble amyloid beta concentrations may not completely reflect the levels in the brain parenchymal. Postmortem tissue lysates analyses may provide a more direct measurement of the amyloid beta content in the brain [135]. It can be hypothesized that soluble amyloid beta might contribute to the development of MBI in the early stages of AD, while plaques might be related to the NPS of AD at later stages. Nevertheless, future studies are needed in order to elucidate the distinct roles of soluble and insoluble amyloid beta levels, combining both CSF and PET imaging evidence.

Possible reasons for the partially contradictory findings of the abovementioned studies could be the limited power in the studies with small sample sizes, the different methods used for the assessment of AD neuropathology (blood, CSF, PET, postmortem neuropathological studies), as well as cognitive decline (clinically meaningful progression and functional decline, defined as MCI or dementia, versus worsening of cognitive performance on neuropsychological tests).The different settings (population-based vs. specialist memory clinics) are also very important, since selection bias (educational level, etc.) might affect study results. Furthermore, the neuropsychological tools used for the assessment of cognitive function also play a crucial role, since some tests might not be able to detect subtle changes in relatively short time periods, including the MMSE. The different follow-up periods of longitudinal studies might also affect the results, since shorter periods might not be sufficient for the detection of significant cognitive, NPS or biomarker changes. The different methods for the characterization of MBI is also another important issue. The use of NPI or NPI-Q provides less specificity compared to MBI-C, mainly because of the shorter time reference period (1 month versus 6 months), leading to the inclusion of transient behavioral changes and reactive conditions due to stressful events. In addition, the NPI and NPI-Q have been constructed for patients with dementia, and some items are not applicable to the targeted population in the context of MBI. In addition, optimal cut-off points for the characterization of MBI (including MBI-C and NPI) remain to be determined, especially for individuals with normal cognition. In this context, a specific, yet unknown, threshold of MBI-C or NPI, corresponding to “more severe MBI” might exert greater effects on the relationship between MBI and cognitive decline.

In order to elucidate the temporal relationship between amyloid deposition, tau accumulation and the development of NPS in preclinical stages of AD, longitudinal studies in individuals with normal cognition or MCI combining multimodal imaging techniques, such as functional MRI (fMRI) for brain connectivity and PET imaging for amyloid burden, might be particularly helpful. In this regard, MBI has been recently linked to connectivity alterations in fMRI, as observed in the early stages of AD [136].

The recently approved anti-Aβ monoclonal antibodies for early stages of AD, aducanumab and lecanemab, have shown promising results in clinical trials by targeting specific forms of amyloid in the brain [137]. Real-world clinical evidence combined with the use of AD neuroimaging and/or fluid biomarkers regarding the effects of these agents on NPS in patients with early stages of AD will offer novel insights into the role of amyloid pathology in MBI.

The lack of associations between MBI and amyloid pathology might be at least partially attributed to the presence of cases with other neuropathologies, either alone or in addition to AD. Importantly, concurrent pathologies are often detected in patients with neuropathologically confirmed AD, including infarcts or other vascular pathology, Lewy bodies, as well as transactive response DNA-binding protein (TDP-43) pathology [138]. A postmortem study has demonstrated that subcortical arteriosclerotic leukoencephalopathy and Lewy body pathology, but not the burden of AD neuropathology, were related to psychotic symptoms in patients with AD [139]. MBI has also been associated with increased white matter hyperintensities volume among individuals with MCI [140]. Hence, other neurobiological mechanisms independent of the amyloid- or tau-related pathways, such as vascular or a-synuclein-mediated mechanisms, might be implicated in the relationship between MBI and cognitive decline [24].

Neurofilament light chain (NfL) levels reflect axonal damage and can be also used as a marker of neurodegeneration. Although NfL is not AD specific, higher NfL levels in individuals with AD have been correlated with more rapid cognitive decline and conversion to dementia. Interestingly, MBI has been linked to a longitudinal increase in plasma NfL levels [141], suggesting that MBI might reflect a faster neurodegenerative process in AD. The relationship between MBI and tau pathology seems to follow Braak staging as discussed above, further supporting this hypothesis. However, longitudinal clinical evidence is needed to clarify this relationship.

Further investigation of the genetic factors associated with MBI would also offer valuable insights into its pathophysiology. In this context, as recently reviewed by our team, the APOE genotype and the MS4A genetic locus have been linked to the MBI domain of affective dysregulation, EPHA1 and BIN1 with psychotic manifestations, *ZCWPW1* with psychosis and social inappropriateness, as well as *NME8* with lack of motivation [21]. In addition, emerging evidence highlights the potential relationship between polygenic risk scores (PRSs) for AD dementia and MBI [142]. In particular, future studies should also aim to investigate the possible association between MBI as a whole or specific MBI domains and pathway-specific PRSs, including those related to Aβ deposition and clearance [21]. This knowledge will aid in elucidating the role of amyloid pathology in MBI development and progression to dementia.

Gender-specific relationship between MBI and amyloid or tau pathologies should be also explored. In this regard, it has been shown that women with AD-related psychosis displayed higher levels of p-tau in the superior frontal gyrus compared to women with AD without psychosis, while men did not exhibit this relationship [143]. In addition, treadmill exercise has been associated with gender-specific behavioral and neurobiological effects on mouse models of AD. In particular, although this intervention was linked to a lower Aβ42/Aβ40 ratio in the brain of males and females, only males displayed improved GABA-A receptor function and inhibition of oxidative stress [144]. Hence, regional-specific relationships between MBI and both tau and amyloid pathology should be further explored separately in men and women.

Interestingly, tau pathology has been linked to NPS and particularly depression in both early- and late-onset AD [48]. Compared to late-onset, early-onset AD has been shown to display a higher burden of tau and amyloid pathology [145]. Although by definition MBI is observed in subjects older than 50 years of age, it would be interesting for future studies to investigate potential differences between early- and late-onset AD in the context of the preclinical and prodromal stages of the disease. Given the differences in the clinical phenotypes of early- versus late-onset AD including NPS [146], this clarification would also help towards our better understanding of the underlying pathophysiology.

Furthermore, brain and cognitive reserve, often linked to intracranial volume and education, respectively, have been consistently shown to affect the clinical trajectory of AD, particularly in terms of cognitive impairment. In particular, higher education and increased intracranial volume have been correlated with better cognitive performance at baseline across the AD spectrum, while a faster cognitive decline has been observed in highly educated demented patients [147]. In this context, a newly defined concept of “behavioral reserve” or “neuropsychiatric reserve” might bee also useful in regard to neurodegeneration, indicating a resilience to the development of behavioral disturbances in the context of MBI, especially in the presence of amyloid and/or tau pathology. Early stressful life events, personality traits, the degree of social engagement throughout life, as well as the quality of interpersonal relationships and support might be considered as potential contributing factors.

Future research efforts will aid in our deeper understanding of the relationship between amyloid and tau pathology with MBI, the development of novel targeted therapeutic approaches, and the improvement of clinical outcomes of individuals at risk of developing AD. Given the fluctuating nature of depressive symptoms, regular assessments of mood changes during the follow-up are needed. Clinical studies with proper MBI assessment ideally with MBI-C, large sample sizes and longer follow-up periods are needed to effectively investigate the complex role of AD-related neuropathology in MBI development and its progression to dementia. For this purpose, data sharing through collaborative research initiatives should be encouraged. In addition, the use of advanced computational methods including machine learning techniques in large epidemiological studies may significantly contribute to the identification of biomarkers and neurobiological mechanisms associated with MBI or progression to dementia, facilitating the early detection of individuals at risk. Subsequent in vitro and in vivo studies may shed more light on the cellular mechanisms and molecular pathways involved.

## 10. Conclusions

The elucidation of the underlying neurobiology of MBI would aid in our deeper understanding of the preclinical phases of AD, facilitate the effective design of relative clinical trials, and ultimately improve clinical outcomes of patients at the earliest stages of the disease.

## Figures and Tables

**Table 1:** Clinical studies investigating the role of amyloid and tau pathology in mild behavioral impairment (MBI).

Reference	Study Type	Aims of the Study	Study Population	MBI Assessment	Amyloid and/or Tau Pathology Assessment	Main Findings
[54] (data from ADNI)	Longitudinal	To investigate the modulatory effects of AD pathology on the relationship between MBI and cognitive impairment	1129 non-demented participants (543 with MCI, 586 with normal cognition)	NPI-Q	Amyloid: Amyloid PET Tau: tau PET	Cross-sectionally: MBI was related with increased amyloid burden At baseline, Aβ partially mediated the relationship between MBI and cognitive impairment (global cognition, memory, executive function, language) No significant association was found between MBI and tau burden, and neurodegeneration assessed by FDG PET Longitudinally: MBI was associated with a more rapid increase in amyloid burden and an increased risk of cognitive decline
[58] (data from BIOCARD study)	Longitudinal	To investigate whether amyloid burden and WMH affect the relationship between NPS and cognitive decline in non-demented individuals	193 cognitively intact individuals	NPI (and GDS)	Amyloid: Amyloid PET	NPS were unrelated to amyloid or WMH burden Amyloid burden did not affect the relationship between NPS and cognitive decline
[61] (data from ADNI)	Longitudinal	To investigate the relationship between MBI and p-tau181, cognitive performance and incident dementia	571 non-demented individuals (201 with normal cognition, 370 with MCI)	NPI MBI defined as NPI > 0 in both visits (baseline and year 1) NPS-not-MBI: defined as NPI > 0 in 1 visit No-NPS: NPI = 0 in both visits	Tau: Plasma p-tau181	Cross-sectionally, MBI (but not NPS-not-MBI) was related to higher levels of plasma p-tau181, compared to no-NPS. Longitudinally, MBI was related to higher levels of p-tau181, as well as cognitive decline (memory, executive function)
[62]	Cross-sectional	To investigate the relationship between the integrity of the locus coeruleus-norepinephrine system, AD stage, and NPS, independent of amyloid or tau burden	118 individuals with normal cognition, 44 with MCI, 28 with AD dementia	MBI assessed by MBI-C	Tau and amyloid PET (locus coeruleus-norepinephrine system integrity was assessed by neuromelanin-sensitive MRI)	In tau-positive participants, higher LC signal was correlated with more severe NPS, independent of tau or amyloid burden This correlation was driven mainly by the MBI domain of impulse dyscontrol
[63] (data from the BioFINDER-2 study)	Cross-sectional	To investigate the relationship between MBI and tau pathology	50 cognitively normal individuals with amyloid pathology (defined by the CSF Aβ42/Aβ40 ratio)	MBI assessed by MBI-C	Tau: tau PET and CSF p-tau181	Increased signal in entorhinal cortex/hippocampus (Braak region I–II) in tau PET and p-tau181 levels in the CSF were correlated with higher MBI-C scores These relationships remained significant after adjustments for cognitive function
[55] (data from the TRIAD cohort	Cross-sectional	To investigate the relationship between MBI and AD biomarkers (amyloid, tau, neurodegeneration)	96 cognitively normal individuals	MBI assessed by MBI-C	Tau and amyloid PET	MBI-C score was associated with increased amyloid burden in amyloid PET No significant associations were detected between tau pathology and MBI-C scores
[20] (data from the NACC)	Longitudinal	To investigate whether MBI and MBI domains could predict the progression to clinical and neuropathologically confirmed AD	11.372 cognitively normal individuals at baseline, 300 of which with postmortem data	NPI-Q	Postmortem samples and neuropathological examination (amyloid plaques, NFTs)	MBI could predict the progression to both the clinical and the neuropathologically confirmed diagnosis of AD [defined by intermediate or high NIA-AA ADNC (ABC score)]. Psychosis was the MBI domain with the largest effect on this association, followed by social inappropriateness, impulse dyscontrol, decreased motivation and affective dysregulation MBI could not predict the progression to AD with none or low NIA-AA ADNC
[64]	Cross-sectional	To investigate the differences in NPS between preclinical/prodromal AD versus prodromal PDD/DLB	103 non-demented individuals	NPI-Q	Amyloid: amyloid PET (dopaminergic neurodegeneration also assessed by DAT-SPECT)	MBI-psychosis scores were higher in Group 1 (amyloid-positive, abnormal DAT-SPECT) compared to Group 5 (normal cognition, amyloid-negative, normal DAT-SPECT) MBI total scores and MBI impulse dyscontrol scores in Group 4 (MCI unlikely due to AD, normal DAT-SPECT) were higher compared to Group 5
[9] (data from two cohorts: ADNI and MEMENTO)	Cross-sectional and longitudinal	To investigate cross-sectioally and longitudinally the relationship between MBI in individuals with MCI and ADCSF biomarkers (amyloid, p-tau, total-tau)	510 individuals with MCI (352 from ADNI, 158 from MEMENTO), with available NPI or NPI-Q data in two consecutive visits	NPI or NPI-Q MBI defined as NPI > 0 in both visits NPS-not-MBI: defined as NPI > 0 in 1 visit No-NPS: NPI = 0 in both visits	Amyloid and tau: CSF (Aβ42, Aβ40, p-tau, t-tau, and Aβ42/Aβ40, p-tau/ Aβ42, t-tau/Aβ42 ratios)	In ADNI, cross-sectionally: Compared to no-NPS, MBI was related to reduced Aβ42, reduced Aβ42/40, increased p-tau, t-tau, p-tau/Aβ42, and t-tau/Aβ42. Compared to no-NPS, NPS-not-MBI was related only to reduced Aβ42/40. In ADNI, longitudinally: MBI was related to reduced Aβ42 and Aβ42/40, increased p-tau, t-tau, p-tau/Aβ42 and t-tau/Aβ42. NPS-not-MBI was related to increased t-tau. In MEMENTO, cross-sectionally: Compared to no-NPS, MBI was related to reduced Aβ42, increased p-tau, p-tau/Aβ42, and t-tau/Aβ42. No differences were detected between NPS-not-MBI and no-NPS. In MEMENTO, longitudinally: MBI was related to increased p-tau, p-tau/Aβ42, t-tau/Aβ42
[29] (data from ADNI)	Cross-sectional	To investigate the relationship between MBI and plasma Ab42/Ab40	139 non-demented individuals (86 with normal cognition, 53 with MCI)	NPI	Amyloid: Plasma Ab42/Ab40	Higher MBI score was related to lower Ab42/Ab40 Affective dysregulation, but neither impulse dyscontrol nor impaired drive/motivation were related to Ab42/Ab40
[65] (data from ADNI)	Longitudinal	To investigate the relationship between MBI and cortical tau pathology in early-stage AD	442 non-demented individuals (283 with normal cognition, 157 with MCI)	NPI MBI defined as NPI > 0 in both visits NPS-not-MBI: defined as NPI > 0 in 1 visit No-NPS: NPI = 0 in both visits	Amyloid and Tau: amyloid and tau PET	In the group with amyloid pathology, MBI (but not NPS-not-MBI) was related to tau uptake in Braak I (right and left entorhinal cortex) and Braak III(left and right parahippocampus, amygdala, posterior cingulate gyrus, fusiform, and lingual gyrus) regions. In the group without amyloid pathology, MBI was not related to tau in the Braak I, but was negatively associated with tau in Braak III region. Braak II was not included in the study for technical reasons

Abbreviations: AD, Alzheimer’s disease; ADNI, Alzheimer’s disease Neuroimaging Initiative; DAT-SPECT, dopamine transporter single photon emission computed tomography; FDG, 18F-fluorodeoxyglucose; MBI, mild behavioral impairment; MBI-C, mild behavioral impairment checklist; MCI, mild cognitive impairment; NIA-AA, National Institute on Aging/Alzheimer’s Association; NACC, National Alzheimer’s Coordinating Center; NFTs, neurofibrillary tangles; NPI, Neuropsychiatric Inventory; NPI-Q, Neuropsychiatric Inventory Questionnaire; PET, positron-emission tomography; PDD, Parkinson’s disease dementia; DLB, dementia with Lewy bodies; SUVRs, standardized uptake value ratios; WMH, white matter hyperintensities; TRIAD, Translational Biomarkers of Aging and Dementia; p-tau181, plasma phosphorylated tau at threonine 181.

## Data Availability

Not applicable.
